# Contribution of rare variation to degenerative orthopedic diseases

**DOI:** 10.1016/j.ocarto.2026.100767

**Published:** 2026-02-25

**Authors:** Christian Anker-Hansen, Eric Manderstedt, Christina Lind-Halldén, Christer Halldén, Bengt Zöller

**Affiliations:** Center for Primary Health Care Research, Department of Clinical Sciences, Lund University and Region Skåne, Jan Waldenströms gata 35, Malmö, 20502, Sweden

**Keywords:** Osteoporosis, Genetics, Exome sequencing, Molecular epidemiology, Osteoarthritis, Musculoskeletal diseases

## Abstract

**Objective:**

Degenerative orthopedic diseases (DODs) such as osteoporosis, osteoarthritis, spondylosis, spinal stenosis, and disc herniation are common disorders. Both common and rare genetic risk factors may contribute to DODs, but few large-scale whole-exome sequencing studies elucidating the contribution of rare variations to DODs have been published. The updated version of the Astra Zeneca portal (https://azphewas.com) was used to access gene collapsing analysis of rare variations for DODs.

**Method:**

One published UK Biobank portal: the updated Astra Zeneca portal based on whole genome sequencing (N = 484,111), was used to access gene collapsing analysis of rare qualifying variants (QVs) for fourteen DODs. A conservative threshold (p ≤ 5 × 10^−8^) was used to decrease the risk of spurious associations. Odds ratios (ORs) with 95 % confidence intervals (CIs) were estimated.

**Results:**

One previously osteoporosis-linked gene (*LRP5*) was identified *(*p-value = 3.0 × 10^−13^, OR = 1.55 (95%CI 1.38–1.73)). Two other significant genes were identified: the *TET2* gene that was linked to dorsalgia (p-value = 8.3 × 10^−9^, OR = 1.54 (95%CI 1.33–1.77)) and the *SMAD6* gene that was associated with spinal stenosis (p-value = 1.7 × 10^−9^, OR = 2.70 (95%CI 2.02–3.61)) and other spondylopathies (p-value = 9.0 × 10^−11^, OR = 2.41 (95%CI 1.89–3.07)). Among controls 1.10 % were carriers of *LRP5* QVs, 0.29 % of controls carried *TET2* QVs, and 0.20 %–0.21 % of controls carried *SMAD6* QVs.

**Conclusion:**

One established osteoporosis gene (*LRP5*) and two other DODs genes (*TET2* and *SMAD6*) were identified using gene collapsing analyses. Only a small number of genes reached the chosen significance threshold under the current phenotype definitions and analytic framework.

## 1 Introduction

Degenerative orthopedic diseases (DOD) such as osteoarthritis, osteoporosis, and back pain due to spondylosis, spinal stenosis, and intervertebral disc degeneration are common and complex disorders where both genetic and acquired factors are involved [1,2]. Genome wide association studies (GWAS) have identified an increasing number of common variants associated with several DODs [[3], [4], [5]]. Exome sequencing may reveal causal rare variants for common complex disorders [[6], [7], [8], [9], [10], [11]]. However, most efforts for complex disorders have relied on GWAS, which focuses on common variants. Whole-exome sequencing (WES) or whole genome sequencing (WGS) studies can reveal the contribution of rare variants to common diseases such as DODs. However, only few WES studies of DODs have been published [[6], [7], [8]]. We used a published but updated UK biobank portal (https://azphewas.com/) [9] to access gene collapsing analysis of rare variation for DODs.

## 2 Methods

The Astra Zeneca portal published by Wang et al. originally reported the correlation between rare protein-coding variants and 17,361 binary phenotypes using WES data derived from 269,171 UK Biobank participants (https://azphewas.com/) [9]. The last version of the UK Biobank 500k WGS (v2) Public has been updated and is based on 484,111 whole-genome sequenced (WGS) individuals (https://azphewas.com/). The smaller Genebass portal (https://app.genebass.org/) that contains 394,841 UK biobank was not used [10]. All used data are publicly available, and ethical approval or informed consent was therefore not applicable. The gene-based collapsing approach, in which variants that satisfy specific criteria (qualifying variants, QV) are binned together as equivalent, is a simple but effective approach to identifying rare-variant contributions to disease [[9], [10], [11]]. The number of cases and controls with rare qualifying mutations in the gene are thereafter compared. Only the model with the lowest p-value of 12 tested models was selected from the Astra Zeneca portal (https://azphewas.com) [9]. In order not to obtain false positive associations, we used a more conservative p-value often used in GWAS studies (*p* ≤ 5 × 10^−8^) [[4], [5], [6], [7],[12], [13], [14]] instead of the commonly used p-value for WES studies (p-values <0.05/20,000 genes = 2.5 × 10^−6^) [[3], [4], [5],[9], [10], [11]]. In fact, the prespecified used threshold of 5 × 10^−8^ is close to the Bonferroni corrected p-value for WES studies (<0.05/20,000 genes/14 tested phenotypes/12 tested models = 1.5 × 10^−8^). The results are exploratory and should be interpreted under the chosen significance framework. We only show the union of the three-digit ICD-10 codes (International Classification of Diseases 10th Revision) [[9], [10], [11]]. Fourteen different DODs were included (Table 1): Osteoarthritis (M15-M19); back diseases (M47-M54), and osteoporosis with and without pathological fracture (M80, M81). Bioinformatic analysis was performed using Genecards (https://www.genecards.org) [12], OMIM (https://www.omim.org/) [13], and the GWAS catalog (https://www.ebi.ac.uk/gwas/) [14].

**Table 1:** Results of gene collapsing analysis of rare variants for Degenerative orthopedic diseases (DODs) according to ICD-10 codes in the Astra Zeneca Portal (https://azphewas.com/) [9]. Union was used to define phenotypes for https://azphewas.com (updated version Dataset: UK Biobank 500k WGS (v2) Public). Only genes meeting the prespecified *p* ≤ 5 × 10^−8^ threshold are shown. Data was accessed 20251026. Odds ratios (ORs) are given with 95 % confidence intervals (CIs). For significant variants number of individuals and percentage with qualifying variants (QV) among cases and controls are given. Only the model with the lowest p-value of 12 tested models was selected from the Astra Zeneca portal (https://azphewas.com) [9].

ICD-10 codes	Cases (QV n, %)	Controls (QV n, %)	Gene, p-value, OR (95%CI)
M15 Polyarthrosis	15,448	122,880	Not significant
M16 Coxarthrosis	27,444	137,961	Not significant
M17 Gonarthrosis	43,791	155,123	Not significant
M18 Arthrosis of first carpometacarpal joint	2848	109,830	Not significant
M19 other arthrosis	86,172	132,789	Not significant
M47 spondylosis	34,472	138,266	Not significant
aM48 other spondylopathies	16,513, (82, 0.50 %)	148,889, (308, 0.21 %)	*SMAD6* 9.0 × 10^−11^, OR = 2.41 (1.89–3.07)
aM48.0 spinal stenosis	9,939, (54, 0.54 %)	154,052, (311, 0.20 %)	*SMAD6* 1.7 × 10^−9^, OR = 2.70 (2.02–3.61)
M50 Cervical disc disorders	4781	157,699	Not significant
M51 other intervertebral disc disorders	26,819	151,286	Not significant
M53 other dorsopathies not elsewhere classified	6041	119,393	Not significant
bM54 dorsalgia	75,189, (333, 0.44 %)	144,041, (416, 0.29 %)	*TET2* 8.3 × 10^−9^, OR = 1.54 (1.33–1.77)
M80 osteoporosis with pathological fracture	2526	113,109	Not significant
bM81 osteoporosis without pathological fracture	24,886, (422, 1.70 %)	97,889, (1,080, 1.10 %)	*LRP5* 3.0 × 10^−13^, OR = 1.55 (1.38–1.73)

Abbreviations: WGS = whole genome sequencing; ICD-10 = International Classification of Diseases 10th Revision; For Astra Zeneca portal (https://azphewas.com), the lowest p-value of the 12 tested models is shown [9].

a UR: Model includes non-synonymous, predicted to be damaging (REVEL score ≥0.25), and Ultra-rare (minor allele frequency ≤0.00005 within the cohort and absent from gnomAD) (https://azphewas.com/) [9].

b ptvraredmg: Model includes non-synonymous, predicted to be damaging (REVEL score ≥0.25), PTVs (protein truncating variants) must be moderately rare (minor allele frequency ≤0.001 within the cohort and within gnomAD), and non-PTVs must be rare (minor allele frequency ≤0.00025 within the cohort and gnomAD global_raw ≤0.00005 and popmax ≤0.0005) (https://azphewas.com/) [9].

## 3 Results

In Table 1, only genes meeting the prespecified *p* ≤ 5 × 10^−8^ threshold are shown. Only the model with the lowest p-value of 12 tested models was selected from the Astra Zeneca portal (https://azphewas.com) [9]. One previously osteoporosis-linked genes (*LRP5*) were identified (Table 1) [[12], [13], [14]]. The only other two significant genes were the *TET2* gene that was linked to dorsalgia (M54) and the SMAD6 gene that was associated with spinal stenosis (M48.0) and other spondylopathies (M48). (Table 1). No other DODs or genes were associated in the rare variant gene collapsing analysis (Table 1). Among controls 1.10 % were carriers of *LRP5* QVs, 0.29 % of controls carried QVs for *TET2*, and 0.20 %–0.21 % of controls carried QVs for *SMAD6* (Table 1).

No *SMAD6* variants, i.e. rare or common, were linked to M48 or M48.0 in the variant analyses in the Astra Zeneca portal (https://azphewas.com) [9] or the GWAS catalog (https://www.ebi.ac.uk/gwas/) [14]. Nor were any rare or common *TET2* variants linked to M54 in the variant analyses of the Astra Zeneca portal (https://azphewas.com) [9] or the GWAS catalog (https://www.ebi.ac.uk/gwas/) [14]. However, several *LRP5* variants were linked to M81 in the variant analysis of the Astra Zeneca portal (https://azphewas.com) [9] and the GWAS catalog (https://www.ebi.ac.uk/gwas/) [14]. Thus, common variants are unlikely to contribute to the associations with *SMAD6* and *TET2* but may contribute to the association with the *LRP* gene.

The biological context and prior evidence as the result of bioinformatic search are presented in Table 2. The *TET2* gene is linked to Myelodysplastic syndrome and Clonal hematopoiesis of indeterminate potential (CHIP) [12,13,15]. CHIP genes like *TET2*, *DNMT3A*, and *ASXL1* have not previously been linked to DODs [[12], [13], [14]] Mutations in CHIP genes like *TET2* may cause an increase in proinflammatory cytokines, resulting in chronic inflammation and immune imbalance and have been linked to many disorders [15]. The *SMAD6* gene is linked to aortic valve disease, craniosynostosis, and radioulnar synostosis but not previously to DODs [[12], [13], [14]]. The *SMAD6* gene encodes for a protein that belongs to the SMAD family of proteins, which are related to Drosophila ‘mothers against decapentaplegic' and *C. elegans* [12,13]. Among its related pathways are gene expression (transcription) and transforming growth factor beta (TGF-beta) receptor signaling in skeletal dysplasias [12,13]. *SMAD6* is an inhibitory Smad (i-Smad) that negatively regulates signaling downstream of type I transforming growth factor-beta and acts as a mediator of TGF-beta and Bone Morphogenetic Protein anti-inflammatory activities [12,13].

**Table 2:** Biological context and prior evidence. Bioinformatic search of the three genes associated with Degenerative orthopedic diseases (DODs) in the gene collapsing analysis of rare variants in the published UK Biobank portal (https://azphewas.com/ [9]. The GWAS catalog (https://www.ebi.ac.uk/gwas/) [14], OMIM (https://www.omim.org/) [13], and Genecards (https://www.genecards.org) [12] were searched for relevant diseases and pathways.

Genes	GWAS catalog^12,14^	Genecards/OMIM^12,13^	Pathway analysis^12^
*SMAD6*	Bone tissue density	Aortic valve disease	Gene expression (transcription)
	body height	Craniosynostosis	TGF-beta receptor signaling in skeletal dysplasias
		Radioulnar synostosis	
*TET2*	body height	Immunodeficiency 75 Myelodysplastic syndrome.	Meiosis Gene expression (transcription)
*LRP5*	Bone tissue density	Osteoporosis-pseudoglioma syndrome	Alzheimer's disease and miRNA effects ncRNAs involved in Wnt signaling in hepatocellular carcinoma
	body height	Osteopetrosis, Autosomal dominant	
	heel bone mineral density	Bone mineral density variability	
		Endosteal hyperostosis	
		Exudative vitreoretinopathy	
		Polycystic liver disease with/without kidney cysts	

Abbreviations: GWAS = genome-wide association study; OMIM=Online Mendelian Inheritance in Man [13]. For a complete list of all GWAS associations, the reader is referred to the original bioinformatic resources [[12], [13], [14]].

## 4 Discussion

The updated version of the portal, UK Biobank 500k WGS (v2), based on 484,111 genomes [9] identified one well established osteoporosis gene (*LRP5*) and two other DODs genes (*SMAD6* and *TET2)*. However, the present study does not suggest that rare variation is a common contributor to common degenerative orthopedic diseases, though QVs in the *SMAD6* gene are strong risk factors for spinal stenosis and other spondylopathies (Table 1). Thus, more common variants have been associated with DODs, especially osteoarthritis and osteoporosis [[3], [4], [5]]. There are no overt connections between the *SMAD6* and *TET2* genes and DODs but due to their involvement in multiple biological processes including regulation of inflammation, it is plausible that the UK biobank findings are correct [[12], [13], [14], [15]] (Table 2).

A strength of UK biobank is the large number of sequenced exomes, i.e. 484,111 in the last version (https://azphewas.com/) [9]. A limitation is the lack of validation studies in independent cohorts and more diverse populations concerning our findings, which will require large studies. It is, however, reassuring that one previously osteoporosis-linked gene (*LRP5*) [[12], [13], [14]] was identified. Other limitations are the lack of experimental evidence, and the use of a 3-digit ICD-10 code for the diagnosis of DODs in the UK Biobank without knowledge of the exact diagnostic procedures. Moreover, owing to the large number of comparisons, some associations might be spurious; we therefore applied a more conservative threshold (*P* ≤ 5 × 10^−8^) than the genome-wide threshold commonly used for WES studies (2.5 × 10^−6^) [[3], [4], [5],[9], [10], [11]]. On the other hand, potential candidates may therefore be discarded.

A limitation regarding *TET2* is that no sensitivity analysis has been done with exclusion of established CHIP-driver mutations in *TET2* to see if the association with dorsalgia remains. It is therefore possible that the finding is driven by age-related clonal hematopoiesis rather than a specific mechanism involving back pain.

The use of different models for the rare variant analysis in the portal (https://azphewas.com/) [9] is both a strength and a limitation. The use of 12 different models and selection of the model with the lowest p-value from the Astra Zeneca portal increases the chance of detecting more associations but also the risk for spurious associations. Another limitation is that rare variants can be highly population specific and findings derived from predominantly European datasets may not be generalizable to other ethnic groups. Some variants may also be limited to selected patients and families.

In conclusion, rare variations were significantly associated with DODs in the *LRP5*, *TET2*, and *SMAD6* genes. Thus, rare variation contributes to DODs in the general population, though only a small number of genes reached the chosen significance threshold under the current phenotype definitions and analytic framework. Future rare variant analysis studies need to incorporate diverse populations or include selected patients and families with DODs.

## Author contributions

All authors made substantial contributions to the conception and design of the work, and the interpretation of data. CAH and BZ drafted the original manuscript. All tother authors substantially contributed to the revision of the manuscript drafts. All authors have approved the submitted version of the manuscript and agreed to be accountable for any part of the work.

## Funding

This work was supported by a grant awarded to Dr Bengt Zöller by ALF-funding from 10.13039/501100009780Region Skåne, and by the 10.13039/501100004359Swedish Research Council. The funders had no role in the study.

## Conflicts of interest

None.

## References

[1] Kelsey J.L., Hochberg M.C. (1988). Epidemiology of chronic musculoskeletal disorders. Annu. Rev. Publ. Health.

[2] Vo N., Niedernhofer L.J., Nasto L.A., Jacobs L., Robbins P.D., Kang J. (2013). An overview of underlying causes and animal models for the study of age-related degenerative disorders of the spine and synovial joints. J. Orthop. Res..

[3] Richards J.B., Rivadeneira F., Inouye M., Pastinen T.M., Soranzo N., Wilson S.G. (2008). Bone mineral density, osteoporosis, and osteoporotic fractures: a genome-wide association study. Lancet.

[4] arcOGEN Consortium, arcOGEN Collaborators, Zeggini E., Panoutsopoulou K., Southam L., Rayner N.W., Day-Williams A.G., Lopes M.C. (2012). Identification of new susceptibility loci for osteoarthritis (arcOGEN): a genome-wide association study. Lancet.

[5] Kim H.A., Heo S.G., Park J.W., Jung Y.O. (2018). Novel genetic variants associated with lumbar spondylosis in Koreans : a genome-wide association study. J. Korean Neurosurg. Soc..

[6] Styrkarsdottir U., Helgason H., Sigurdsson A., Norddahl G.L., Agustsdottir A.B., Reynard L.N. (2017). Whole-genome sequencing identifies rare genotypes in COMP and CHADL associated with high risk of hip osteoarthritis. Nat. Genet..

[7] Skarp S., Kämäräinen O.P., Wei G.H., Jakkula E., Kiviranta I., Kröger H. (2018 Aug 29). Whole exome sequencing in Finnish families identifies new candidate genes for osteoarthritis. PLoS One.

[8] Cilia C., Friggieri D., Vassallo J., Xuereb-Anastasi A., Formosa M.M. (2022). Whole genome sequencing unravels new genetic determinants of early-onset familial osteoporosis and low BMD in Malta. Genes.

[9] Wang Q., Dhindsa R.S., Carss K., Harper A.R., Nag A., Tachmazidou I. (2021). Rarevariant contribution to human disease in 281,104 UK Biobank exomes. Nature.

[10] Karczewski K.J., Solomonson M., Chao K.R., Goodrich J.K., Tiao G., Lu W. (2022). Systematic single-variant and gene-based association testing of thousands of phenotypes in 394,841 UK Biobank exomes. Cell Genom..

[11] Zöller B., Manderstedt E., Lind-Halldén C., Halldén C. (2025). Rare-variant collapsing analyses of asthma in the UK biobank. Respir Investig.

[12] Stelzer G., Rosen N., Plaschkes I., Zimmerman S., Twik M., Fishilevich S. (2016). The GeneCards suite: from gene data mining to disease genome sequence analyses. Curr. Protoc. Bioinform..

[13] Hamosh A., Amberger J.S., Bocchini C., Scott A.F., Rasmussen S.A. (2021). Online Mendelian inheritance in man (OMIM®): Victor McKusick's magnum opus. Am. J. Med. Genet..

[14] Sollis E., Mosaku A., Abid A., Buniello A., Cerezo M., Gil L. (2023). The NHGRI-EBI GWAS catalog: knowledgebase and deposition resource. Nucleic Acids Res..

[15] Zhang J.L., Tong S.L., Zhuang Q.Q., Jin S.J., Li J.Q., Sun J. (2025). Clonal hematopoiesis of indeterminate potential: a multisystem hub bridging hematopoietic dysfunction with non-hematopoietic diseases. Mil Med Res.

